# CLINICAL PREDICTORS OF FAMILIAL DEPRESSION IN HAN CHINESE WOMEN

**DOI:** 10.1002/da.20878

**Published:** 2011-11-07

**Authors:** Lina Wang, Dongdong Qiao, Yihan Li, Liwei Wang, Jianer Ren, Kangmei He, Jing Sun, Zhoubing Wang, Tian Tian, Ce Chen, Lei Yang, Jian Hu, Hong Deng, Qian Wang, Keqing Li, Jiyang Han, Han Rong, Zhaoyu Gan, Hong Yang, Pingliang Zhou, Jiyang Pan, Cong Zhou, Yanping Cui, Libo Song, Yuzhang Zhu, Ying Li, Xueyi Wang, Lanxian Ye, Wei Liang, Yunchun Chen, Qingjun Tang, Jing Guan, Shenxun Shi, Kenneth S Kendler, Jonathan Flint, Lanfen Liu

**Affiliations:** 1 Shandong Mental Health CenterJinan, Shandong, People's Republic of China; 2 Wellcome Trust Centre for Human GeneticsOxford, United Kingdom; 3 Fudan University affiliated Huashan HospitalShanghai, People's Republic of China; 4 Shanghai Jiao Tong University School of Medicine affiliated Shanghai Mental Health CentreShanghai, People's Republic of China; ^5^Shanghai Tongji University affiliated Tongji HospitalShanghai, People's Republic of China; 6 Nanjing Brain HospitalNanjing, Jiangsu, People's Republic of China; 7 No. 4 Affiliated Hospital of Jiangsu UniversityZhenjiang, Jiangsu, People's Republic of China; 8 Tianjin Anding HospitalHexi District, Tianjin, People's Republic of China; 9 No. 1 Hospital of Medical College of Xian Jiaotong UniversityXi'an, Shaanxi, People's Republic of China; 10 No.1 Hospital of Zhengzhou UniversityZhengzhou, Henan, People's Republic of China; ^11^No. 1 Mental Health Center Affiliated Harbin Medical UniversityNangang District, Haerbin, Heilongjiang, People's Republic of China; 12 Mental Health Center of West China Hospital of Sichuan UniversityWu Hou District, Chengdu, Sichuan, People's Republic of China; 13 Beijing Anding Hospital, Capital Medical UniversityXicheng District, Beijing, People's Republic of China; 14 Hebei Mental Health CenterBaoding, Hebei, People's Republic of China; 15 ShengJing Hospital of China Medical UniversityHeping District Shenyang, Liaoning, People's Republic of China; 16 Shenzhen Kangning HospitalLuo Hu, Shenzhen, Guangdong, People's Republic of China; 17 No. 3 Affiliated Hospital of Zhongshan UniversityGuangzhou, Guangdong, People's Republic of China; 18 No.1 Hospital of Shanxi Medical UniversityTaiyuan, Shanxi, People's Republic of China; 19 Mental Hospital of Jiangxi ProvinceNanchang, Jiangxi, People's Republic of China; 20 The First Affiliated Hospital of Jinan UniversityGuangzhou, Guangdong, People's Republic of China; 21 Wuhan Mental Health CenterWuhan, Hubei, People's Republic of China; 22 No.3 Hospital of Heilongjiang ProvinceBeian, Heilongjiang, People's Republic of China; 23 Jilin Brain HospitalSiping, Jilin, People's Republic of China; 24 The First Hospital of China Medical UniversityShenyang, Liaoning, People's Republic of China; 25 Dalian No. 7 People's Hospital & Dalian Mental Health CenterDalian, Liaoning, People's Republic of China; 26 The First Hospital of Hebei Medical UniversityShijiazhuang, Hebei, People's Republic of China; 27 Lanzhou University Second Hospital, Second Clinical Medical College of Lanzhou UniversityLanzhou, Gansu, People's Republic of China; 28 Psychiatric Hospital of Henan ProvinceXin Xiang, Henan, People's Republic of China; 29 The Fourth Military Medical University affiliated Xijing HospitalXi'an, Shaanxi, People's Republic of China; 30 No. 4 People's Hospital of LiaochengLiaocheng, Shandong, People's Republic of China; 31 Guangzhou Brain Hospital/Guangzhou Psychiatric HospitalGuangzhou, Guangdong, People's Republic of China; 32 Virginia Commonwealth University, Department of Psychiatry, Virginia Institute for Psychiatric and Behavioral GeneticsRichmond, Virginia

**Keywords:** major depression, family history, symptom, life events

## Abstract

**Background:**

A number of clinical features potentially reflect an individual's familial vulnerability to major depression (MD), including early age at onset, recurrence, impairment, episode duration, and the number and pattern of depressive symptoms. However, these results are drawn from studies that have exclusively examined individuals from a European ethnic background. We investigated which clinical features of depressive illness index familial vulnerability in Han Chinese females with MD.

**Methods:**

We used lifetime MD and associated clinical features assessed at personal interview in 1,970 Han Chinese women with DSM-IV MD between 30–60 years of age. Odds Ratios were calculated by logistic regression.

**Results:**

Individuals with a high familial risk for MD are characterized by severe episodes of MD without known precipitants (such as stress life events) and are less likely to feel irritable/angry or anxious/nervous.

**Conclusions:**

The association between family history of MD and the lack of a precipitating stressor, traditionally a characteristic of endogenous or biological depression, may reflect the association seen in other samples between recurrent MD and a positive family history. The symptomatic associations we have seen may reflect a familial predisposition to other dimensions of psychopathology, such as externalizing disorders or anxiety states. Depression and Anxiety 0:1–6, 2011. © 2011 Wiley-Liss, Inc.

## INTRODUCTION

Major depression (MD), a common, costly, and frequently recurrent disorder, is both etiologically and clinically heterogeneous. Risk for MD most likely arises from a combination of biological, environmental, and genetic factors.[1, 2] Furthermore, affected individuals are likely to vary in their phenotypic characteristics such as age at onset, severity, duration, patterns of symptoms, recurrence, and level of impairment.

A number of studies document that familial factors strongly influence the risk of developing MD.[3–8] According to the most recent meta-analysis of high-quality family studies, first-degree relatives of patients with MD have a two to threefold higher lifetime risk for depression compared to relatives of controls.[9] Individuals with depression, however, likely differ in their underlying familial risk. The consistent finding that familial factors influence risk for MD,[3, 4, 10] has prompted a search to identify clinical correlates of familial susceptibility.[3, 4, 7, 10–19]

Investigations have found a number of clinical features that are related to the risk of illness in relatives, including age at onset,[3, 4, 6, 7, 11, 13, 18] recurrence,[4, 6, 12, 13] impairment[12, 13] and the number and pattern of depressive symptoms.[4, 13] However, as Sullivan et al. point out,[9] many of these studies have relatively small sample sizes, do not always control for cohort effects and their results may be biased due to sample selection criteria. Studies that do not suffer from these methodological concerns confirm that familial risk is reflected in some clinical features, including age of onset,[19, 20] the number of MDD episodes experienced, and the number of DSM-IV criteria endorsed.[19]

However, prior studies have exclusively examined individuals from a European ethnic background. While there has been some interest in determining whether and why rates of depression vary between cultures,[21, 22] much less work has been devoted to exploring the extent to which clinical features diverge.[23, 24] Lee et al. point out that in psychiatric nosology “symptoms unique to non-Western cultures are generally neglected.”[25] We lack information on the extent to which clinical features differ between studies. Importantly, the pattern and similarities are likely to cast light on the origins and nature of MD.

The purpose of our study was to determine whether the clinical features of MD that reflect a high familial vulnerability to illness are a basic aspect of depression, or whether these clinical features differ across ethnic groups. We asked to what extent it was possible to predict, from the clinical features reported in earlier studies, the liability for MD in family members of female depressed probands from a Han Chinese background.

## MATERIAL AND METHODS

### SUBJECTS

The data for this study were drawn from the ongoing China, Oxford and VCU Experimental Research on Genetic Epidemiology (CONVERGE) study of major depression (MD). These analyses were based on a total of 1,970 cases recruited from 53 provincial mental health centres and psychiatric departments of general medical hospitals in 41 cities in 19 provinces and four central cities: Beijng, Shanghai, Tianjin and Chongqing and 2,597 controls who were recruited from patients undergoing minor surgical procedures at general hospitals or from local community centers. All cases and controls were female and had four Han Chinese grandparents. Cases and controls were excluded if they had a pre-existing history of bipolar disorder, any type of psychosis or mental retardation. Cases were aged between 30 and 60 years, had two or more episodes of MD, with the first episode occurring between 14 and 50 years and had not abused drug or alcohol before the first episode of MD. Controls were chosen to match the region of origin of cases, were aged between 40 and 60 years, had never experienced an episode of MD and were not blood relatives of cases. An older minimal age of controls was used to reduce the chances that they might have a subsequent first onset of MD. The mean age (and *SD*) of cases and controls in the data set was 45.1 (8.8) and 47.7 (5.5), respectively.

All subjects were interviewed using a computerized assessment system. All interviewers were trained by the CONVERGE team for a minimum of 1 week in the use of the interview. The interview includes assessment of psychopathology, demographic and personal characteristics, and psychosocial functioning. Interviews were tape-recorded and a proportion of them was listened to by the trained editors who provided feedback on the quality of the interviews.

The study protocol was approved centrally by the Ethical Review Board of Oxford University and the ethics committee in participating hospitals in China.

### MEASURES

The diagnoses of MD was established with the Composite International Diagnostic Interview (CIDI) (WHO lifetime version 2.1; Chinese version), which classifies diagnoses according to the Diagnostic and Statistical Manual of Mental Disorders (DSM-IV) criteria.[26]

The history of lifetime major depression in the parents and siblings was assessed using the Family History Research Diagnostic criteria.[27]

The interview was originally translated into Mandarin by a team of psychiatrists in Shanghai Mental Health Centre with the translation reviewed and modified by members of the CONVERGE team.

The interview was fully computerized into a bilingual system of Mandarin and English developed in house in Oxford called SysQ. Skip patterns were built into SysQ. Interviews were administered by trained interviewers and entered offline in real time onto SysQ, which was installed in the laptops. Once an interview was completed, a backup file containing all the previously entered interview data could be generated with database compatible format. The backup file, together with an audio recording of the entire interview, was uploaded to a designated server currently maintained in Beijing by a service provider. All the uploaded files in the Beijing server were then transferred to an Oxford server quarterly.

### STATISTICAL ANALYSIS

All statistical analyses were carried out with the SPSS package (version 17.0; SPSS Inc., Chicago, IL). We estimated means and standard deviations (*SD*) of all relevant demographic and clinical characteristics of major depression. The dependent variable was a positive family history of MD that we defined as the presence of a lifetime diagnosis of MD in one or more of the patient's first-degree relatives (that is, parents or siblings). We coded family history as follows: if one or more of the patient's first-degree relatives had a lifetime history of depression we coded 1, if not we coded 0. A total of 20 clinical characteristics of MD were examined included age at onset of depression, the number of lifetime episodes, impairment during the worst episode, duration of the longest episode, the sum of DSM-IV A criteria for MD that were met, the presence of individual DSM-IV A criteria, the respondent's report about the cause of their most severe episode of MD (1=caused by life events; 0=have no reason), and other symptoms of MD. When taken one variable at a time, χ^2^-test was used to compare categorical variables and logistic regression analysis was performed to investigate FH and continuous variables. When examining all positive variables, forward and backward stepwise logistic regression was performed to find the best predictors of familial MD.

## RESULTS

The sample comprised 1,970 patients with a lifetime history of MD. A total of 69 (3.5%) people did not finish the family history section correctly and were therefore excluded, leaving a total of 1,901 patients. Of these 1,901 patients, 624 (32.8%) reported that at least one of her first-degree relatives had a lifetime history of MD.

We examined the ability of our clinical indices to predict the presence of MD in one or more first-degree relatives of patients. When examined one variable at a time (Table 1), a report by the patient that their most severe MD episode was not attributable to a known cause significantly predicted risk for MD in the relatives. We refer to this as “MD episode with no known cause.” Endorsement of two DSM-IV criteria (A7: “worthlessness” and A8: “trouble concentrating”) and two other symptoms (irritable/angry and anxious/nervous) also predicted risk of MD in the relatives with *P*-values <.05. However, only one of these features (feeling anxious/nervous) remained significant when correcting for multiple testing (*P*<.0025 correcting for 20 tests). Of note, early age at onset in the patients and number of endorsed A criteria for MD were associated in our data with an increased risk for MD in relatives. However, the effects were small and only at a trend level of significance (*P*=.07 and .08).

**TABLE 1:** Prediction of MD in the relatives from the clinical features of MD in a proband of the family

Phenotype	*P* value	OR	95% CI
*Clinical features*			
Onset age	.07	0.99	0.98–1.00
Number of episodes	.33	1.00	1.00–1.01
Impairment	.73	0.99	0.93–1.06
Longest episode	.93	1.00	1.00–1.00
Number of A criteria	.08	1.07	0.99–1.15
MD episode with no known cause	.006	1.34	1.09–1.66
*Symptoms*			
DSM “A” criteria			
A-1 depressed mood	.09	3.59	0.81–15.84
A-2 loss of interest	.40	1.35	0.67–2.72
A-3 weight change	.13	1.30	0.92–1.84
A-4 insomnia	.24	1.34	0.83–2.16
A-5 slowed down/restlessness	.20	1.24	0.89–1.72
A-6 fatigue	.15	1.33	0.90–2.00
A-7 worthlessness	.04	1.40	1.01–1.94
A-8 trouble concentrating	.02	2.34	1.13–4.84
A-9 thoughts of death	.99	1.00	0.80–1.25
Feeling irritable/angry	.006	0.92	0.87–0.98
Feeling hopeless	.63	0.99	0.93–1.05
Crying a lot	.14	0.96	0.91–1.01
Feeling helpless	.05	0.92	0.85–1.00
Feeling anxious/nervous	.0006	0.85	0.78–0.93

OR, *P*-values, and 95% confidence intervals (95% CI) are shown for clinical characteristics of MD, including the nine DSM-IV symptom criteria. ORs greater than one indicate that the clinical feature or symptom is more common in probands with one or more first-degree relatives with a history of MD. ORs less than one indicate that the clinical feature or symptom is less common in probands with one or more first-degree relatives with a history of MD. OR, odds ratio; MD, major depression; DSM-IV, Diagnostic and Statistical Manual of Mental Disorders.

We next included all five individually significant variables in stepwise logistic regression analysis to determine the unique predictors of familial depression. The results of this analysis showed that “MD episode with no known cause,” a lack of symptoms of irritability or anger, and a lack of symptoms of anxiety or nervousness predict greater risk in relatives (Table 2).

**TABLE 2:** Significant predictors of MD in the relatives from the clinical features of MD in a proband

Variable	*P*	OR	95% CI
Episode of MD with no known precipitant	.017	1.066	1.01–1.12
Irritable/angry	.05	0.944	0.89–1.00
Anxious/nervous	.001	0.857	0.78–0.94

OR, *P*-values, and 95% confidence intervals (95% CI) are shown for including all significant predictors (*P*<.05) obtained from Table 1. ORs greater than one indicate that the clinical feature or symptom is more common in probands with one or more first-degree relatives with a history of MD. ORs less than one indicate that the clinical feature or symptom is less common in probands with one or more first-degree relatives with a history of MD. OR, odds ratio; MD, major depression.

## DISCUSSION

In this study, we asked whether familial risk for MD influenced the clinical features and symptoms of MD in a large sample of Chinese women. Examined one at a time, we found five features and symptoms of MD that predict a history of MD in one or more relatives. Probands who reported no known precipitant to their worst episode and symptoms of worthlessness and trouble concentrating and who denied symptoms of irritability/anger or anxiety/nervousness had a significantly increased risk of reporting a parent or sibling with lifetime MD. Two other clinical features (early age at onset and number of A criteria) and one symptom (feeling helpless) were also associated with risk for MD in relatives, but only at a trend level. In a multivariate analysis, “MD episode with no known cause” and lack of symptoms of feeling irritable/angry and anxious/nervous were found to be significant and unique predictors of familial depression. We found no evidence that recurrence predicted risk of MD in relatives.

Our findings partly replicate those found in the United States and European studies. Sullivan et al.[9] reviewed the literature on clinical indices of familial aggregation and found that recurrence of MD predicted familial aggregation in all studies. Five of the eight community-based studies they considered sreported that early age at onset of MD predicted familial aggregation; a recent study of 13,000 twin pairs concluded that age of onset had a modest inverse relationship to the risk of illness in relatives.[19] Our findings of a weak relationship of early age at onset and family history thus may be consistent with these prior studies in Western populations.

Sullivan et al.[9] found that one of the three studies reported that familial risk for MD in relatives was associated with number of reported symptoms, again broadly consistent with our finding of a modest statistical trend in this direction. Specific MD symptoms associated with familiality have been less well studied. Kendler et al. studied a cohort of twins and found that biological symptoms reflecting appetite/sleep changes were predictive of MD in the co-twin.[14]

One of our strongest findings is the association between no precipitating stressor and the worst episode. This has not, to our knowledge, been examined in previous studies of symptoms as indices of familial risk. This result is intriguing because the lack of a precipitating stressor is traditionally associated with endogenous depression, often considered to be more “biological” and genetic. Furthermore, this finding may be associated with the kindling phenomenon, typically seen in recurrent MD. As reviewed previously,[28, 29] a number of studies have shown that with an increased number of recurrences of MD, the proportion of episodes that occur without precipitating events increases. Thus, our finding of a strong relationship of “endogenous” episodes being associated with a family history of MD may indirectly reflect the association seen in other samples between recurrent MD and positive family history. Furthermore, Kendler et al.[30] have shown that among subjects with a high genetic risk for MD, they begin their course of depressive illness “pre-kindled,” that is with a higher proportion of onsets unassociated with stressful events. These results are directly analogous to our findings here.

To our surprise, we found two symptoms experienced during the depressive episode to predict a lower risk for MD in relatives: irritability/anger and anxiety/nervousness. We are not aware of prior studies that have examined the relationship between familial loading for MD and these symptoms of an MD episode. While it is premature to speculate extensively in the absence of replicating data, these symptoms may reflect a familial predisposition to other dimensions of psychopathology, such as externalizing disorders or anxiety states. Unfortunately, the only form of psychopathology that we assessed in relatives in our study was MD, so we are unable to pursue these hypotheses further.

One pertinent issue here is the cultural continuity of symptoms. Kleinman has argued persuasively that some aspects of depression are experienced differently, for example somatic experiences,[25, 31] and depression may not be separated from anxiety or fear.[32] It is possible therefore that the pattern of symptoms we have observed may reflect a culturally specific experience of depression.[23]

Our findings are subject to a number of limitations. First, our patients were identified through hospitals; we did not include depressed people living in the community. Our findings may not generalize to Han Chinese males, or Chinese females who do not seek treatment. Second, the clinical features of MD were reported retrospectively and were not obtained by direct interview with relatives. Human memory is fallible and prior studies have suggested that some clinical features of MD are recalled with only moderate reliability.[33, 34] Furthermore, mental illness itself may alter the validity of information obtained at interview.[35] We cannot rule out the possibility that biased recall shaped the pattern of results we have observed. Third, our study was carried out with subjects with recurrent MD, so that we are not able to comment on the extent to which our findings will apply to those with a single episode. Finally, we note here that we do not have estimates of inter-rater reliability of diagnoses in cases and in relatives; thus, we caution that there is an unspecified degree of error in estimates of the effect sizes that we observe.
